# Psychological Interventions for Insomnia in Patients with Cancer: A Scoping Review

**DOI:** 10.3390/cancers16223850

**Published:** 2024-11-16

**Authors:** Alyssa Alinda Gonzalez, Gladys Janice Jimenez-Torres, Aline Rozman de Moraes, Yimin Geng, Varsha Pawate, Rida Khan, Santhosshi Narayanan, Sriram Yennurajalingam

**Affiliations:** 1 Department of Palliative Care, Rehabilitation, and Integrative Medicine, The University of Texas MD Anderson Cancer Center, Houston, TX 77030, USA; gjjimenez@mdanderson.org (G.J.J.-T.); arozman@mdanderson.org (A.R.d.M.); vbpawate@mdanderson.org (V.P.);; 2 Research Medical Library, The University of Texas MD Anderson Cancer Center, Houston, TX 77030, USA

**Keywords:** insomnia, cancer, psychological interventions, cognitive behavioral therapy, behavioral therapy, mindfulness, relaxation

## Abstract

Background/Objectives: Insomnia is a frequent and distressing symptom with limited treatment in patients with cancer. Among the available treatments for insomnia, psychological interventions are some of the most assessed. While studies and reviews show promise of interventions improving insomnia in patients with cancer, inconsistent approaches to operationalizing and measuring insomnia combined with the heterogeneity of available treatments render comparisons and synthetization difficult. This review sought to rigorously synthesize psychological interventions for patients with cancer and insomnia by describing current therapies, identifying gaps in the literature, and offering supportive interventions. Methods: A comprehensive search of five databases—Ovid MEDLINE, Ovid Embase, Ovid PsycInfo, EBSCO CINAHL Plus with Full Text, and Cochrane Library—was conducted through 1 August 2024. The review included randomized trials and non-inferiority trials evaluating psychological interventions for patients with various types of cancers. Twenty-one studies were included, and study quality ranged from good to excellent, according to the PEDro scale. Results: The current evidence supports the use of cognitive behavioral therapy (CBT) to treat insomnia over and above psychoeducation alone, mindfulness-based stress reduction, Brief Behavioral Therapy, the Benson Relaxation Technique, progressive muscle relaxation, mindfulness meditation, mind-body bridging, and home-based psychological nursing. Digital, minimal, and internet-based intervention modalities are effective, though professional-delivered CBT proved most effective. Conclusions: CBT remains the most effective psychological intervention for insomnia in this population. Challenges exist regarding the implementation of CBT, so further research is warranted to identify the best psychological interventions for specific settings and subsets of patients with cancer.

## Simple Summary

Insomnia is prevalent in patients with cancer, with rates ranging from 30% to 60%. Addressing insomnia remains important in this population, as it can improve patient quality of life and reduce suffering. However, inconsistent methods of defining insomnia and evaluating potential interventions renders treatment difficult. Furthermore, many factors influence insomnia’s presentation, requiring a specialized and individualized approach to its management for each patient. This review sought to use thorough and strict criteria to assess current psychological intervention for insomnia. To this end, we describe current psychological treatment options for insomnia, highlight gaps in the existing research, and offer suggestions for treating insomnia in patients with cancer.

## 1. Introduction

Insomnia is among the most frequently reported and debilitating sleep disturbances in patients with cancer, affecting their quality of life, emotional well-being, overall survival, and health outcomes [1,2,3]. In this population, insomnia occurs at nearly three times the rate in the general population, with prevalence estimates ranging from 30% to 60%, and varying based on the definition of insomnia, the timing of the assessment, and the measurement methods used [4,5,6]. The high incidence of insomnia in patients with cancer warrants effective interventions to improve the quality of life and alleviate suffering.

Insomnia is defined as difficulty falling asleep and/or maintaining sleep that causes distress and impairs daily functioning [1]. Insomnia is characterized by dissatisfaction with sleep quality or quantity due to difficulty either falling or remaining asleep, waking up earlier than desired, with difficulty resuming sleep, and experiencing non-restful sleep despite having the opportunity to sleep [1,7]. Insomnia predicts and worsens common cancer symptoms such as fatigue, pain, anxiety, and depression, contributing to a harmful feedback loop further impairing daily functioning and overall quality of life [6,8,9].

Previous research identified links between genetic factors and insomnia in individuals with and without cancer. Studies suggest insomnia is associated with dysregulation of hypothalamic-pituitary-adrenal axis activity [10]. Additionally, genetic factors play a significant role in determining individual sleep requirements, accounting for approximately one-third of the variability in susceptibility for insufficient sleep [11,12]. For example, variations in specific CLOCK genes, such as the rs12649507 and rs11932595, correlate with longer sleep duration in European populations [13]. In contrast, among African Americans, the presence of the T allele in the rs2070062 single nucleotide polymorphisms (SNPs) is linked to shorter sleep duration, even after adjusting for confounding variables such as European ancestry, socioeconomic status, body mass index, alcohol consumption, and smoking status [14]. In patients with cancer, the disease itself, along with its location in the body, and patient sex can alter CLOCK gene expression, further influencing sleep regulation [15,16]. Specifically, women with certain genetic variants (e.g., the rs11133391 T/T genotype) and high (expression) levels of miR-192, miR-206, miR-194, and miR-219 demonstrate significantly better survival rates in metastatic colorectal cancer compared to men [17]. This suggests that gender-specific CLOCK gene variants not only influence sleep regulation and insomnia but also survival outcomes. These findings underscore that while genetic variations in CLOCK genes may affect sleep duration across populations, gene-environment interactions may play a crucial role in both the development of and recovery from insomnia.

Specific locations of genes on a chromosome correspond with metabolic or psychiatric traits, suggesting the existence and expression of multiple phenotypes of insomnia [18]. In patients with cancer, genetic predispositions combined with cancer-related variables can increase the risk for insomnia [19]. For example, in patients with breast cancer, polymorphisms in inflammatory genes (interleukin 1 receptor 2 [IL1R2], IL13, and NFKB2) are associated with insomnia [20]. Other studies exploring genetics found that DRD2 CT genotype versus CC and a higher number of chemotherapy cycles are significantly associated with lower Pittsburgh Sleep Quality Index (PSQI) scores [21,22]. A prior study by Irwin and colleagues [23] found that interventions, such as Cognitive Behavioral Therapy for Insomnia (CBT-I), are associated with reduced systemic inflammation and expression of genes encoding proinflammatory mediators. Patients with cancer and insomnia exhibit a high prevalence of psychiatric symptoms, such as depression and anxiety, and physical symptoms, such as pain, fatigue, and tiredness, further complicating its management [24,25,26]. Effectively managing insomnia and related symptoms remains crucial for comprehensive cancer care, as it can decrease sleep disturbances, lower the probability of developing physical symptoms, and enhance quality of life [26,27,28].

Psychological interventions show potential in treating insomnia across populations and are the most investigated treatments for insomnia and sleep quality. Published systematic reviews and meta-analyses investigating the benefits of psychological therapies in patients with cancer show that cognitive-behavioral therapy for insomnia (CBT-I), mindfulness-based therapies (MBTs), and other mind-body techniques improve sleep parameters and reduce psychological distress [29,30]. CBT-I proves beneficial for treating insomnia in early-stage patients with cancer, with substantial evidence supporting its benefits in improving sleep quality and general well-being [31]. Similarly, MBTs and Mindfulness-Based Stress Reduction (MBSR) demonstrate potential in mitigating the severity of insomnia and reducing stress in patients with cancer, suggesting that these therapies help regulate arousal levels [32,33,34]. Despite positive outcomes, limited systematic reviews exist evaluating current psychological interventions for insomnia in patients with cancer. The unique needs of this population require targeted research to establish the effectiveness of these interventions. While reviews exploring insomnia exist, many seek to evaluate the prevalence of insomnia, insomnia in patients with certain complications, digital interventions, approaches to studying sleep disturbances, and specific treatments for insomnia [2,35,36,37,38,39,40,41,42].

Furthermore, none of the studies use rigorous criteria to define the interventions, incorporate an approach to generalize findings or evaluate the impact of providing clinically relevant benefits over time (even after the completion of study interventions). This scoping review aims to systematically synthesize psychological interventions for insomnia in patients with cancer. To this end, we describe current therapies, identify any gaps in the current research, and offer healthcare practitioners strategies for improving insomnia management in clinical practice for this susceptible group.

## 2. Methods

Findings of this scoping review are reported in accordance with the guidelines of the Preferred Reporting Items for Systematic Reviews and Meta-Analyses (PRISMA) statement, and the PRISMA Extension for Scoping Reviews was utilized [43].

### 2.1. Studies

The focus of this scoping review included randomized controlled trials and noninferiority studies comparing psychological interventions with placebo, standard of care, or other intervention for insomnia in patients with any cancer (early cancer, cancer patients undergoing treatment, advanced cancer, cancer patients post-treatment). Our review encompasses studies between 1 January 2000 to 1 August 2024 to ensure the inclusion of enough recent and relevant studies to identify patterns in the research and interventions. Published reviews assessing psychological interventions have not found studies prior to 2000 [42,44]. Additional inclusion and exclusion criteria are shown in Table 1.

### 2.2. Interventions

Included studies evaluated psychological interventions for the management of insomnia in patients with cancer. These interventions consisted of psychotherapy/psychoeducation, meditation, cognitive behavioral therapy (CBT), mindfulness-based stress reduction (MBSR), behavioral therapy, and sleep management training. Studies without a psychotherapeutic rationale or theory supporting the intervention were excluded. Intervention descriptions are shown in Table 2.

### 2.3. Outcome Measures

Studies were required to have “insomnia” as a primary outcome of interest. To this end, those evaluating insomnia using a valid and acceptable sleep (insomnia) measure, such as the Pittsburgh Sleep Quality Index (PSQI), Insomnia Severity Index (ISI) Epworth Sleepiness Scale (ESS), Consensus Sleep Diary (CSD), European Organisation for Research and Treatment of Cancer Quality of Life Questionnaire—30-item (EORTC-QLQ-30) sleep, Edmonton Symptom Assessment System (ESAS) sleep item, Patient-Reported Outcomes Measurement Information System (PROMIS-sleep), or any other scale that assesses both presence and/or severity of sleep disturbance) met the criteria for inclusion. Articles only assessing insomnia using a subjective sleep measure, that were unpublished, or that were ongoing trials were excluded.

### 2.4. Literature Search Strategy

We conducted a systematic search of Ovid MEDLINE, Ovid Embase, Ovid PsycInfo, EBSCO CINAHL Plus with Full Text, and Cochrane Library databases for publications in the English language from 1 January 2000 to 1 August 2024. The concepts searched include “neoplasm”, “cancer”, “sleep”, “sleep wake disorders”, “insomnia”, “wakefulness”, “sleeplessness”, “sleep deprivation”, “sleep disturbance”, “sleep disorder”, “sleep disruption”, “sleep latency”, “sleep efficiency”, “sleep initiation”, “sleep duration”, “sleep quality”, “total sleep time” and “daytime sleepiness” etc. Both subject headings and keywords were utilized. The terms were combined using AND/OR Boolean Operators. The search results were limited to clinical trials, prospective studies, and surveys. Animal studies, in vitro studies, conference abstracts, case reports, and retrospective studies were removed from the search results. The complete search strategies are detailed in Tables S1–S5.

### 2.5. Data Collection and Analysis

An institutional librarian (YG) conducted the initial search prior to article screening. Duplicates, articles in a language other than English, and articles irrelevant to the focus of this review were removed. Six review authors (AG, SY, ARM, SN, RK, and VP) independently screened the abstracts and titles to determine eligibility for inclusion in the review. Full-text articles were collected and screened (by ARM, AG, SY, RK, VP, and GJJT) for rigorous review and those determined to meet inclusion criteria were selected for data extraction. At each stage of the review, two reviewer responses were required for consensus. Disagreements of the reviewer’s findings were discussed with the input of the Principal Investigator (SY).

### 2.6. Data Extraction and Management

Review author AG extracted data from the articles independently. The authors met to discuss articles needing further clarification when needed.

### 2.7. Quality of the Evidence

The Physiotherapy Evidence Database (PEDro) scale was utilized in the screening of the articles to determine the quality of the studies evaluating insomnia interventions for patients with cancer (by AG and ARM).

## 3. Results

**Figure 1:**
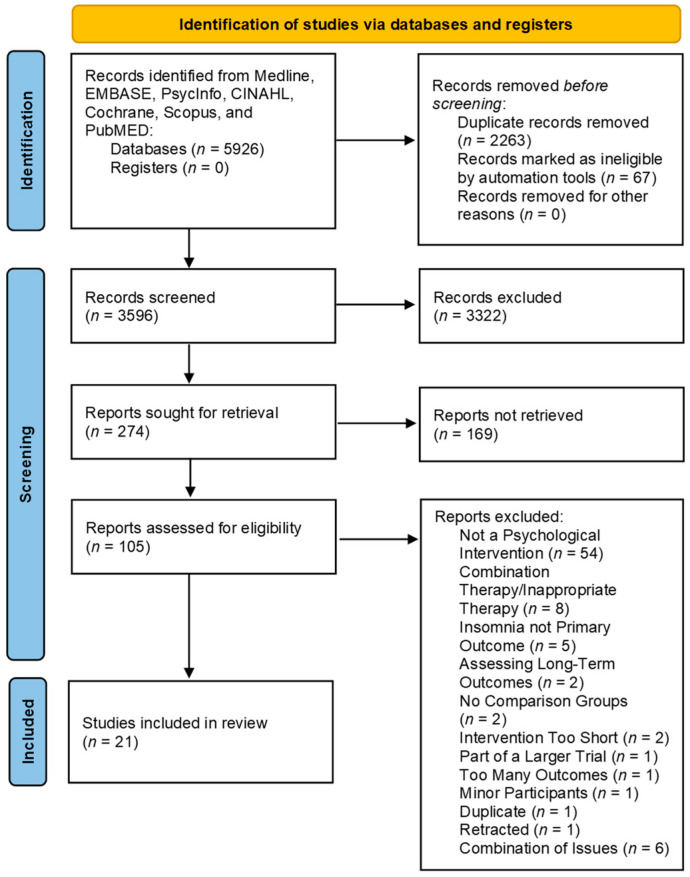
PRISMA Flowchart.

The PRISMA Diagram in Figure 1 shows identified and excluded studies at each step of the review. Two searches of five databases in 2023 and 2024 identified a total of 5926 potential articles relevant to insomnia. After excluding duplicates (*n* = 2263) and others marked as ineligible by automated tools (*n* = 67), a total of 3596 moved onto the screening process. Of these, 3322 records were excluded, 274 were sought for retrieval but 169 were not retrieved, leaving 105 full-text articles that were assessed for eligibility. Following the review, 84 articles were excluded, resulting in 21 randomized controlled trials fulfilling the eligibility criteria for inclusion.

## 4. Description of Included Studies

Data from the included articles were extracted. The 21 randomized controlled trials included data from 1957 participants (1089 intervention/psychological interventions and 868 controls/non-psychological interventions) who were analyzed. Most studies were conducted in the United States (*n* = 8), followed by Canada (*n* = 5), South Korea (*n* = 1), United Kingdom (*n* = 1), China (*n* = 2), Denmark (*n* = 1), Turkey (*n* = 2), and Iran (*n* = 1). Study characteristics are depicted in Table 3.

## 5. Participants

On average, the age of participants was 55.7 years, and 75.4% were women. About half of the studies (*n*  =  11) reported the racial composition of the sample. Most participants in these studies were White, with an average proportion of 89% across samples. Most studies consisted of patients with mixed (*n* = 10) and breast cancers (*n*  =  8), others were lung (*n*  =  2) and hypopharyngeal carcinoma (*n*  =  1). The primary cancer stage across studies was stage 1 (*n*  =  7) followed by stage 2 (*n* = 5). Two additional studies evaluated early-stage patients (stages 1 and 2 equally), and one examined advanced-stage patients (stages 3 and 4). Two studies assessed patients with no metastasis, and four did not describe the stages of the participants. About half of the studies included cancer survivors (*n*  =  11), and 10 assessed patients undergoing active primary cancer treatment. Demographics are shown in Table 3.

## 6. Psychological Interventions

Details of the interventions are provided in Table 4. Out of the 21 total studies reporting the effectiveness of psychological interventions, 12 utilized variations of CBT (one used CBT, six used CBT-I, one used CBT for insomnia and pain, one compared professional-delivered vs. self-delivered video CBT-I, one evaluated minimal CBT-I, one explored a digital, app-based CBT-I, and a final study assessed internet-delivered CBT-I). Two incorporated brief behavioral treatment for insomnia, two used progressive muscle relaxation, one evaluated the Benson Relaxation Technique, one utilized mindfulness-based stress reduction, one assessed home-based psychological nursing, one explored mindfulness-based cognitive therapy, and a final study compared mindfulness meditation vs. mind-body bridging.

## 7. Control Group and Non-Psychological Interventions

There were a variety of controls used for comparisons within the controlled trials. Information about the treatment groups is included in Table 4.

## 8. Outcomes

Various measures were used to evaluate insomnia. The Insomnia Severity Index (ISI) was utilized in 8 studies, while the PSQI was used in 7 studies. Five studies utilized a variation of a sleep diary: two used a 14-day Sleep Diary, one used a “Sleep Diary”, one incorporated the 3-day Sleep Diary, and one used the Sleep–Wake Diary. The Medical Outcomes Study Sleep Scale (MOS-SS) was used in one study. Out of the various assessments measuring insomnia, the ISI followed closely by the PSQI was the most widely utilized tool.

## 9. Quality of the Evidence

We utilized the PEDro scale to evaluate the methodological quality of psychological interventions on insomnia in patients with cancer which are reflected in Table 4. Findings show a range of scores between 6 and 9, with an overall average of 7.10 across all included studies. Seven studies scored a 6, 6 studies scored a 7, 7 scored an 8, and one study scored a 9. Overall, 20 studies were of good quality, and one study was of excellent quality.

## 10. Discussion

In this review, we found 21 out of 105 eligible studies that investigated psychological treatments for insomnia. Among them, cognitive behavioral therapy via various modalities, including in-person, video format, digital app, and self-delivered, as well as using focus groups or individual sessions was most evaluated. Of the 12 CBT interventions, 3 were delivered using video format. The primary outcome measures used in most studies were the ISI and PSQI. The longer-term impact of the intervention after completion of the study was evaluated by most studies (*n* = 13); however, these results were not the focus of our study. Adherence to the intervention was not evaluated in most studies.

CBT was the most evaluated psychological intervention in our study, and findings support its position as the gold standard treatment for insomnia in not only the general population but also for patients with cancer [66]. Our rigorous and comprehensive evaluation of several CBT modalities highlights their effectiveness in reducing insomnia symptoms, yet high attrition rates indicate potential burdens for some patients [67,68]. Limited access to CBT for insomnia hinders widespread implementation, though alternative delivery models, such as stepped-care approaches and prehabilitation clinics show promise for expanding access in populations like veterans and patients with breast cancer [69,70].

As found in previous studies, our review identified a selection bias. Most studies included participants who were predominantly younger, who had a diagnosis of breast cancer, and who had completed cancer treatment or patients with no evidence of disease. The impact of patient age, cancer type, stage, and treatment type were not explored. There were no serious adverse events reported using these interventions. Based on the data, all treatments were found to be effective, with group CBT and in-person CBT preferable.

Recent studies included in this review suggest that brief behavioral therapy, Mindfulness-Based Stress Reduction, progressive muscle relaxation, and the Benson Relaxation Technique are feasible, efficacious, and reasonable alternatives to CBT; however, all studies show heterogeneity in terms of primary outcome measure, number of sessions, and comparative arms. Limited details about adherence to the intervention and facilitators to success were reported. The study quality as measured by the PEDro scoring varied between 6 and 9. Most studies included either cancer survivors or patients with early stages of cancer. Limited studies were conducted in patients with advanced cancer, those on active treatment, and in men. Other limitations include limited reporting of adverse events or concerns or other patient-reported challenges in utilizing these interventions. These limitations impact the generalization of our study findings to other populations not often studied. Therefore, further studies are needed to understand (a) how long the effects of each psychological intervention for insomnia last after the intervention is stopped, (b) the impact of psychological interventions on insomnia in other patients with cancer who are distressed or have advanced cancer, (c) comparisons between other psychological, pharmaceutical, or non-pharmaceutical interventions, (d) meaningfulness of sleep score improvements on patients’ quality of life, functioning, and secondary outcomes, and (e) strategies other than CBT due to potential challenges with this intervention, such as time demands and symptom burden often faced by patients [71]. Furthermore, research investigating biological factors of insomnia warrants further investigation into health disparities and their impact on insomnia identification and treatment.

The challenge with interpreting the results is influenced multifold, notably (1) by the outcome measure which is derived from defining the sleep problem, (2) the criteria determining if a patient with cancer benefits from the intervention, and (3) bias. While we found differences in efficacy between the different psychological interventions, variations in outcome measures may not accurately capture the true benefit of the interventions which may then limit the utilization of potentially useful interventions in the future. Furthermore, the sample bias as well as the predominance of CBT-I intervention studies limit generalization which is consistent with other reviews [31,72,73,74].

Differences in the approach to defining sleep health versus sleep problems contribute to difficulties in operationalizing the expected outcome and therefore limits the potential to improve services [67]. Sleep health has been described as a complex and multidimensional pattern of sleep and wakefulness that is tailored to individual, social, and environmental needs, supporting both physical and mental well-being [29]. While most recognized definitions encompass the subjective experience of distress in determining criteria, most clinical scales have unique psychometric properties which may then limit assessment. Other studies evaluating sleep interventions focus exclusively on the measurement of insomnia as a symptom versus a sleep disorder, which contributes to varying prevalence rates between studies [11]. Inconsistent definitions of acute and chronic insomnia, which comprise insomnia syndrome, also result in variable outcomes that should not be compared, given differences in the impact that acute vs. chronic symptoms may have.

There is a gap in the literature regarding therapeutic approaches to improve insomnia in the population with cancer, specifically the type or duration of intervention. An approach that involves classifying factors associated with insomnia into predisposing, precipitating, and perpetuating categories may risk an overly narrow focus, akin to what occurred first, the ‘chicken or the egg’ dilemma. In contrast, interventions aimed at addressing symptom clusters offer an opportunity to shift focus more broadly toward achieved outcomes. The most effective treatment may ultimately use a multimodal approach, often incorporating behavioral therapies such as Cognitive Behavioral Therapy for Insomnia (CBT-I), non-pharmacological strategies, and pharmacological interventions targeting the pathobiology of insomnia in patients with cancer [73,75].

## 11. Conclusions

Psychotherapeutic approaches remain the most effective strategy to improve insomnia for patients with cancer, and existing treatment options may not only help mitigate this syndrome but also its associated symptom burden. However, when insomnia is treated without the consideration of other factors, insomnia may recur. Therefore, addressing insomnia within the context of sleep health promotion and prevention remains vital. Further, effective insomnia treatment may be achieved by tailoring treatment approaches to account for individual phenotypic and biopsychosocial factors. Hence, initiatives should incorporate a personalized approach where both the treatment and outcome measures may be customized for an individual’s pathobiology. Identifying psychological interventions to treat insomnia in patients with cancer across personal and medical demographics and settings remains an important area of exploration. Future research should identify opportunities to modify interventions to promote maximum improvements in insomnia for patients with cancer, explore the impact of health disparities in the assessment and treatment of insomnia, evaluate long-term outcomes of interventions, determine the impact of interventions on a wider range of patients with cancer, and increase comparison studies with psychological, pharmaceutical, and non-pharmaceutical interventions for insomnia.

## Figures and Tables

**Table 1:** Study Inclusion and Exclusion Criteria.

Inclusion Criteria	Exclusion Criteria
English From Ovid MEDLINE, Ovid Embase, Ovid Psych info, CINHAHL, and Cochrane Library databases Psychology Interventions All Cancer Survivors Prospective Studies Sample Size of 30 or More Insomnia as Primary Outcome Use of Valid Insomnia Measure (PSQI, ISI, ESS, CSD, EORTC-QLQ-30 sleep, ESAS sleep item, PROMIS-sleep, etc.) Intervention duration at least 1 week and compared to placebo or another intervention or standard of care	Combination Interventions Prior to 2000 Less than 30 patients enrolled in the study Assessment of sleep disturbances using only treatment toxicity from the Common Terminology Criteria for Adverse Events (CTCAE) or equivalent

**Table 2:** Intervention Descriptions.

Intervention	Description
Cognitive Behavioral Therapy for Insomnia (CBT-I)	A psychological intervention that targets thoughts and behaviors and is targeted toward insomnia as a presenting problem. Treatment focused on sleep education, sleep behavior information, cognitive restructuring, stimulus control, sleep restriction, relaxation, and imagery [45].
Minimal Cognitive Behavioral Therapy for Insomnia (mCBT-I)	A self-administered CBT treatment for insomnia through bibliotherapy format that is accompanied by 3 brief phone consultations. It combines stimulus control, sleep restriction, cognitive restructuring, and sleep hygiene strategies [46].
Digital Cognitive Behavioral Therapy (dCBT)	A modified CBT intervention for insomnia that is digitally based (app). It incorporates stimulus control, sleep restriction, relaxation, and paradoxical intention. Teachings include calculation of sleep efficiency, sleep hygiene, behavioral activation, and recognition of and how to change dysfunctional sleep-related thoughts. It was developed with patient, medical provider, and mental health provider input [47].
Cognitive Behavioral Therapy for Insomnia and Pain (CBT-i.p.)	A CBT intervention that incorporates aspects of insomnia (psychoeducation, sleep hygiene, stimulus control, and sleep restriction) and pain (psychoeducation and activity pacing), as well as relaxation, cognitive restructuring, pleasant activity scheduling, and self-monitoring/homework [48].
Professional-Delivered Cognitive Behavioral Therapy (PCBT-I)	A CBT-I approach delivered by certified psychologists or clinical psychology students is adapted for patients with cancer [49].
Video-Based Cognitive Behavioral Therapy for Insomnia (VCBT-I)	A self-administered CBT-I treatment that incorporates video sessions and booklets to read [49].
Internet-Based Cognitive Behavioral Therapy (iCBT-I)	A fully automated and interactive approach to CBT for insomnia that is delivered via the internet. It was adapted into Danish and incorporates introduction and treatment rationale, sleep restriction, stimulus control, cognitive restructuring, sleep hygiene, and relapse prevention [50].
Brief Behavioral Treatment for Insomnia (BBT-I)	A brief treatment for insomnia based on primary care practices and CBT-I that emphasizes sleep behavior changes and a physiological model of sleep regulation. It also incorporates information about sleep stages and figures to enhance learning [51].
Brief Behavioral Therapy for Cancer-Related Insomnia (BBT-CI)	An approach based on traditional CBT-I but adapted for patients with cancer who are undergoing chemotherapy. It incorporates psychoeducation, stimulus control, discouragement/modification of napping, sleep compression, and chronorehabilitation [52].
Progressive Muscle Relaxation (PMR)	Involves tensing and relaxing large skeletal muscle groups in a systematic way which can relax the body and promote sleep [53].
Benson Relaxation Technique (BRT)	An easily practiced relaxation technique (incorporating breathing and mindfulness) that can treat factors impacting sleep and results in improved vital signs and muscle tension [54].
Mindfulness-Based Stress Reduction (MBSR)	A program that provides psychoeducation about the association between stress and health and teaches meditation and gentle yoga [55].
Home-Based Psychological Nursing	A nursing approach that incorporates psychological principles such as health education and psychological evaluation, psychogenic relaxation, orienting communication with patient and family, education about post-operation care, and facilitated conversations with family [56].
Mindfulness-Based Cognitive Therapy for Insomnia (MBCT–I)	A program based on the stress-and-coping paradigm that encompasses mindfulness meditation techniques, cognitive and behavioral strategies, and stress management [57].
Mindfulness Meditation (MM)	Based on Mindfulness-Based Stress Reduction and includes teaching and reviewing fundamental mindfulness meditation skills (breath awareness, awareness of thoughts and emotions), body scans, walking meditation, and forgiveness meditation [58].
Mind-Body Bridging (MBB)	A mind–body intervention that teaches awareness skills to recognize dysfunctional mind–body states and impaired mental or physical functioning. It also teaches mind–body “mapping” exercises to identify the link between thought patterns and bodily states [58].

**Table 3:** Study Characteristics.

Author, Year, Country	Cancer Type; Treatment Status; Stage	Demographics (Mean Age, % Female, Race)	Randomized *n*; Analyzed *n*;	Psychological Intervention	Treatment Delivery Mode	Number of Sessions; Total Duration of Intervention	Insomnia Scale	PEDro Scale Score
Cognitive Behavioral Therapy Interventions								
Barton 2020, United States [45]	Mixed cancers Active Treatment Stage: I–III, other	Age: ≤50: 14, 51–70: 27; >70: 2 Female: 83% Race: White	93; 81	Cognitive Behavioral Therapy for Insomnia (CBT-I) ^1^	Self-administered through booklets and audio	42 days over 6 weeks	3-Day Sleep Diary—Change in Sleep Onset Latency and Time to Fall Back to Sleep After Awakening	6
Casault 2015, Canada [46]	Mixed cancers Active Treatment and Survivors	Age: 56.90 Female: 92% Race: White	38; 35	Minimal Cognitive Behavioral Therapy for Insomnia (mCBT-I) ^2^	Self-administered through booklets with 3 brief phone consultations with researcher	30 days over 6 weeks	Insomnia Severity Index (ISI) ^3^—All Subscales	9
Chung 2022, South Korea [47]	Mixed cancers Active Treatment Stage: I–IV, relapse or metastasis	Age: 46.50 Female: 86%	57; 57	Digital Cognitive Behavioral Therapy (dCBT) ^4^	Self-administered through digital app, could contact research team for assistance via text or phone	66 days over 10 weeks	Pittsburgh Sleep Quality Index (PSQI) ^5^—Global Score	8
Espie 2008, United Kingdom [59]	Mixed cancers Survivors	Median Age: 60.50 + 58 Female: 69%	150; 128	Cognitive Behavioral Therapy for Insomnia (CBT-I)	Oncology nurse delivered in-person in groups of 4–6	5 sessions over 5 weeks	Sleep Diary (Sleep Onset Latency and Wake After Sleep Onset, Total Sleep Time, and Sleep Efficiency)	6
Garland 2019, United States [60]	Mixed cancers Survivors Stage: 0–IV, unknown	Age: 61.50 Female: 57% Race: White	160; 160	Cognitive Behavioral Therapy for Insomnia (CBT-I)	Professional-delivered in individual in-person sessions	7 sessions over 8 weeks	Insomnia Severity Index (ISI) Total	8
Irwin 2017, United States [61]	Breast Survivors	Age: 59.80 Female: 100% Race: White	90; 90	Cognitive Behavioral Therapy for Insomnia (CBT-I)	Therapist-administered in in-person groups of 7 to 10	12 sessions across 3 months	Pittsburgh Sleep Quality Index (PSQI) Global Score	8
Matthews 2014, United States [62]	Breast Survivors Stage: I–III	Age: 52.51 Female: 100%	60; 56	Cognitive Behavioral Therapy for Insomnia (CBT-I)	Advanced practice nurse-delivered in-person and over the phone	6 sessions over 6 weeks	Sleep-Wake Diary (Sleep Efficiency, Sleep Latency, Total Sleep Time, Wake After Sleep Onset, Number of Nightly Awakenings)	7
Mercier 2018, Canada [63]	Mixed cancers Survivors Stage: 0–III, unknown	Age: 57.10 Female: 78%	41; 41	Cognitive Behavioral Therapy for Insomnia (CBT-I)	Self-administered through video and booklets	6 sessions over 6 weeks	Insomnia Severity Index (ISI) Total	8
Padron 2022, United States [48]	Mixed Active Treatment Stage: I–IV, unknown	Age: 59.40 Female: 100% Race: White	35; 35	Cognitive Behavioral Therapy for Insomnia and Pain (CBT-i.p) ^6^	Therapist-delivered in-person	6 sessions over 6 weeks	14-Day Sleep Diary—Sleep Efficiency and Sleep Quality	8
Savard 2005, Canada [64]	Breast Active Treatment Stage: I–III	Age: 54.09 Female: 100%	58; 57	Cognitive Behavioral Therapy (CBT)	Professional-delivered in in-person groups of 4–6	8 sessions over 8 weeks	Insomnia Severity Index (ISI) Total	7
Savard 2014, Canada [49]	Breast Active Treatment Stage: 0–III, unknown	Age: 54.40 Female: 100%	242; 242	Professional-Delivered Cognitive Behavioral Therapy (PCBT-I) ^7^ Video-Based Cognitive Behavioral Therapy for Insomnia (VCBT-I) ^8^	Professional-delivered through in-person individual sessions and Self-delivered through video and booklets	6 sessions over 6 weeks	Insomnia Severity Index (ISI) Total	7
Zachariae 2018, Denmark [50]	Breast Survivors Stage: 0–III	Age: 53.10 Female: 100%	255; 255	Internet-Based Cognitive Behavioral Therapy (iCBT-I) ^9^	Self-administered via the internet	6 sessions over 9 weeks	Insomnia Severity Index (ISI) Total and Pittsburgh Sleep Quality Index (PSQI) Global Score	7
Brief Behavioral Interventions								
Dean 2020, United States [51]	Non-small cell lung cancer Survivors Stage: I, II	Age: 65.73 Female: 63% Race: White	40; 30	Brief Behavioral Treatment for Insomnia (BBTI) ^10^	Nurse interventionist delivery of a manualized treatment	4 sessions over 4 weeks	14-Day Sleep Diary; 14-day average of the sleep diary sleep efficiency	6
Palesh 2020, United States [52]	Breast Active Treatment Stage: I–IV	Age: 50.13 Female: 100% Race: White	74; 70	Brief Behavioral Therapy for Cancer-Related Insomnia (BBT-CI) ^11^	Professional-delivered through 2 face-to-face and 4 phone call sessions	6 sessions over 6 weeks	Insomnia Severity Index (ISI) Total	8
Progressive Muscle Relaxation								
Sari 2024, Turkey [53]	Mixed Cancers Active Treatment Stage: II, III	Age: 53.5 Female: 36.23%	80; 69	Progressive Muscle Relaxation (PMRE) ^12^	Self-administered through videos after an in-person training session	2 sessions a day for 8 weeks	Pittsburgh Sleep Quality Scale (PSQI) Sleep Quality	7
Turan 2024, Turkey [65]	Lung cancer Active Treatment Stage: I–IV	Age: 61.61 Female: 40.54%	74; 74	Progressive Muscle Relaxation (PMR) ^12^	Self-administered through audio	56 daily sessions over 8 weeks	Pittsburg Sleep Quality Index (PSQI)—All Subscales	7
Benson Relaxation Technique								
Chabok 2023, Iran [54]	Breast cancer Survivors Non-metastatic stages	Age: 47.11 Female: 100%	72; 72	Benson Relaxation Technique (BRT) ^13^	Self-administered through audio	2 months of self-administered Benson’s relaxation	Pittsburgh Sleep Quality Index (PSQI) Global Score	6
Mindfulness-Based Stress Reduction								
Garland 2014, Canada [55]	Mixed cancers Survivors Non-metastatic stages	Age: 59.44 Female: 72% Race: White	111; 72	Mindfulness-Based Stress Reduction (MBSR) ^14^	Professional-delivered in in-person groups of 6–10 for CBT-I and 15 to 20 for MBSR	8 sessions (+ weekend retreat for MBSR group) over 8 weeks	Insomnia Severity Index (ISI) Total	6
Home-Based Psychological Nursing								
Li 2021, China [56]	Hypopharyngeal Cancer Active Treatment Stage: I–IV	Age: 59.35 Female: 6%	140; 140	Home-based psychological nursing	Nurse-delivered in-person	5 sessions over 5 weeks	Pittsburgh Sleep Quality Scale (PSQI) Global Score	6
Mindfulness-Based Cognitive Therapy								
Zhao 2020, China [57]	Breast cancer Survivors Stage: I–III	Age: 53.04 Female: 100%	136; 136	Mindfulness-Based Cognitive Therapy for Insomnia (MBCT–I) ^15^	Therapist-delivered in in-person groups of 8–10	6 sessions over 6 weeks	Insomnia Severity Index (ISI) Total	8
Mindfulness Meditation and Mind Body-Bridging								
Nakamura 2013, United States [58]	Mixed cancers Survivors	Age: 52.60 Female: 75% Race: White	57; 57	Mindfulness Meditation (MM) ^16^ and Mind-Body Bridging (MBB) ^17^	Professional-delivered through in-person weekly group meetings	3 sessions over 3 weeks	Medical Outcomes Study Sleep Scale (MOS-SS)—SPI-II ^18^	6

^1^ CBT-I = Cognitive Behavioral Therapy for Insomnia, ^2^ mCBT-I = Minimal Cognitive Behavioral Therapy for Insomnia, ^3^ ISI = Insomnia Severity Index, ^4^ dCBT = Digital Cognitive Behavioral Therapy, ^5^ PSQI = Pittsburgh Sleep Quality Index, ^6^ CBTi.p. = Cognitive Behavioral Therapy for Insomnia and Pain, ^7^ PCBT-I = Professional-Delivered Cognitive Behavioral Therapy for Insomnia, ^8^ VCBT-I = Video-Delivered Cognitive Behavioral Therapy, ^9^ iCBT-I = Internet-Delivered Cognitive Behavioral Therapy, ^10^ BBT-I = Brief Behavioral Therapy for Insomnia, ^11^ BBT-CI = Brief Behavioral Therapy for Cancer-Related Insomnia, ^12^ PMRE/PMR = Progressive Muscle Relaxation, ^13^ BRT = Benson Relaxation Technique, ^14^ MBSR = Mindfulness-Based Stress Reduction, ^15^ MBCT-I = Mindfulness-Based Cognitive Therapy for Insomnia, ^16^ MM = Mindfulness Meditation, ^17^ MBB = Mind-Body Bridging, ^18^ MOS-SS = Medical Outcomes Study Sleep Scale.

**Table 4:** Study Interventions and Scores.

Author, Year, Country	Study Design	Intervention	Comparator	Control	Improvements in Insomnia (Post-Intervention)	Attrition	Conclusion
Cognitive Behavioral Therapies							
Barton 2020, United States [45]	Phase II Randomized Controlled Trial	CBT-I ^1^ (sleep hygiene, stimulus control, sleep restriction, and bedtime imagery audio) was self-administered daily over the course of 6 weeks.	Placebo	Sleep hygiene, stimulus control, and bedtime short story audio self-administered daily for 6 weeks.	3-Day Sleep Diary Sleep Latency Baseline CBT-I/Imagery: 45 (32.5) Sleep Hygiene/Story Control: 51.7 (41.5) Post-Treatment CBT-I/Imagery: 26.3 (26.4) Sleep Hygiene/Story Control: 30.2 (39) Time to Fall Back to Sleep Baseline CBT-I/Imagery: 23.9 (20.2) Sleep Hygiene/Story Control: 30.8 (31.3) Post-Treatment CBT-I/Imagery: 19.1 (27.4) Sleep Hygiene/Story Control: 23.9 (26.5)	24.73%	CBT-I and Sleep Hygiene were both effective at improving sleep outcomes; however, there were no statistically significant differences between the two arms.
Casault 2015, Canada [46]	Randomized Controlled Trial	Minimal CBT-I was self-administered through bibliotherapy format (one book each week) with 3 consultation phone calls every 2 weeks for a total of 6 weeks.	No Treatment	No intervention	Insomnia Severity Index Baseline mCBT-I ^2^: 12.06 (0.95) No Intervention: 12.11 (1.24) Post-Treatment mCBT-I: 5.32 (0.71) No Intervention: 11.31 (1.30)	7.89%	Minimal CBT-I was more effective than the no treatment control group at improving insomnia outcomes.
Chung 2022, South Korea [47]	Randomized Controlled Trial	dCBT ^3^ was self-administered daily for 10 weeks (66 days) through the HARUToday Sleep Program app.	Placebo and No Treatment	Attention Control: received only cancer-related information or information on how to manage sleep problems for 66 days, for one session per day, excluding weekends. The sleep quality ratings, as well as the reward and prompting system, were the same as in the HARUToday program. Waitlist Control: participants waited for 66 days, during which the intervention and attention control groups used the corresponding programs. There was no further contact between the participants and the researchers.	PSQI ^4^ Global Score Baseline dCBT: 25.16 (4.84) Attention Control: 24.90 (4.73) Waitlist Control: 24.41 (6.27) Post-Treatment dCBT: 15.63 (10.00) Attention Control: 22.05 (5.26) Waitlist Control: 23.82 (6.09)	21.05%	The digital CBT intervention was more effective than the active and waitlist controls in reducing sleep difficulties.
Espie 2008, United Kingdom [59]	Randomized Controlled Trial	CBT-I delivered in 5 weekly 50-min in-person group sessions by G-grade oncology nurses.	Placebo	Treatment as usual	Sleep Onset Latency Baseline, median (IQR) ^5^ CBT: 41.0 (20.3–64.8) TAU ^6^: 27.4 (22.4–50.0) Post-Treatment, median (IQR) CBT: 19.3 (11.9–26.6) TAU: 27 (16.1–52.8) Total Sleep Time Baseline, median (IQR) CBT: 399 (343.3–455.9) TAU: 392 (348–457.9) Post-Treatment, median (IQR) CBT: 426.3 (370.1–456.8) TAU: 409.0 (327.3–453.3) Wake Time after Sleep Onset Baseline, median (IQR) CBT: 62 (40.7–107.5) TAU: 51 (30.5–82.0) Post-Treatment, median (IQR) CBT: 27 (14–57.5) TAU: 51 (33–93.3) Sleep Efficiency Baseline, median (IQR) CBT: 80.4 (69.5–85.8) TAU: 82.4 (74.5–88.5) Post-Treatment, median (IQR) CBT: 89.8 (81.2–94.0) TAU: 82.0 (73.8–89.1)	14.67%	Group CBT for insomnia was more effective than the treatment as usual control group, as it significantly improved sleep onset latency, wake time after sleep onset, and sleep efficiency.
Garland 2019, United States [60]	Randomized Comparative Effectiveness Trial	Acupuncture was administered twice weekly for 2 weeks, then weekly for 6 more weeks, for a total of 10 treatments for 8 weeks.	Active Comparator	CBT-I across 5 weekly sessions followed by two biweekly sessions for 7 weeks total of intervention.	Insomnia Severity Index Change from Baseline to 8 weeks mean (95% CI) Acupuncture: −8.31 (−9.36, −7.26) CBT-I: −10.91 (−11.97, −9.85)	7.50%	CBT-I was more effective than acupuncture at reducing insomnia.
Irwin 2017, United States [61]	Single-Masked, Single-Site, Parallel Group Noninferiority Trial	TCC ^7^ delivered to groups of 7 to 10 in weekly 120-min sessions.	Active Comparator	CBT-I delivered to groups of 7 to 10 across 3 months of 120-min weekly sessions.	Pittsburgh Sleep Quality Index Baseline Tai Chi: 11.2 (0.4) CBT-I: 11.1 (0.4) Post-Treatment Tai Chi: 8.2 (0.4) CBT-I: 7.3 (0.4)	11.11%	CBT-I and Tai Chi Chih improve insomnia outcomes, and Tai Chi Chih was found to be statistically noninferior to CBT-I.
Matthews 2014, United States [62]	Longitudinal Randomized Controlled Trial	Six individual CBT-I sessions delivered weekly for 15–60 min (sessions 1, 2, 3, and 6 for 30–60 min, sessions 4 and 5 for 15–20 min). 1–3, 6 in-person, and 4 and 5 by phone.	Placebo	Six individual BPT ^8^ sessions delivered weekly for 15–60 min (sessions 1, 2, 3, and 6 for 30–60 min, sessions 4 and 5 for 15–20 min). 1–3, 6 in-person, and 4 and 5 by phone.	Sleep Efficiency (%) Baseline CBT-I: 79.09 BPT: 79.92 Change from week 1–6 CBT-I: 9.39 BPT: 5.99 Sleep Latency (minutes) Baseline CBT-I: 36.79 BPT: 25.46 Change from week 1–6 CBT-I: 20.73 BPT: 7.97 WASO ^9^ (minutes) Baseline CBT-I: 38.25 BPT: 40.84 Change from week 1–6 CBT-I: 20.38 BPT: 12.12 TST ^10^ (minutes) Baseline CBT-I: 394.16 BPT: 382.7 Change from week 1–6 CBT-I: 0.37 BPT: 30.96 Awakenings (per night) Baseline CBT-I: 2.46 BPT: 2.84 Change from week 1–6 CBT-I: 0.68 BPT: 0.78	6.67%	Nurse-delivered CBT-I is better than an active placebo at improving sleep variables.
Mercier 2018, Canada [63]	Randomized Controlled Trial	CBT-I was self-administered via video and booklet once a week for 6 weeks.	Active Comparator	Administration of an individualizing aerobic exercise plan across 3 to 5 20–30 min sessions per week with a gradual increase over time to 150 min of aerobic exercise per week.	Insomnia Severity Index Baseline CBT-I: 14.8 (1.1) Exercise: 16.0 (1.3) Post-Treatment CBT-I: 10.3 (1.3) Exercise: 12.1 (1.7)	7.32%	Both CBT-I and exercise improved sleep, though exercise was found to be significantly inferior to CBT-I.
Padron 2022, United States [48]	Randomized Controlled Trial	CBT-i.p ^11^ was administered in individual 90-min weekly sessions across 6 weeks.	Placebo	Psychoeducation was comprised of six weekly 90-min sessions across 6 weeks.	14-Day Sleep Diary Sleep Efficiency Baseline CBTi.p: 81.7 (9.2) Psychoeducation: 76.0 (11.4) Post-Treatment CBTi.p: 88.0 (9.1) Psychoeducation: 81.8 (7.4) Sleep Quality (total score) Baseline CBTi.p: 2.0 (0.5) Psychoeducation: 2.1 (0.5) Post-Treatment CBTi.p: 2.3 (0.6) Psychoeducation: 2.2 (0.6)	22.86%	CBTi.p and psychoeducation improved sleep difficulties; however, CBTi.p was superior.
Savard 2005, Canada [64]	Randomized Controlled Trial	CBT was administered through eight weekly sessions of approximately 90 min, offered in groups of four to six patients.	No Treatment	Wminaitlist Control	Insomnia Severity Index Baseline Mean (95% CI) CBT: 16.15 (14.25, 18.05) Waitlist Control: 13.70 (11.88, 15.52) Post-Treatment CBT: 7.57 (5.59, 9.55) Waitlist Control: 8.56 (6.72, 10.40)	13.79%	CBT was more effective than a waitlist control at improving subjective sleep indices.
Savard 2014, Canada [49]	Randomized Controlled Trial	PCBT-I ^12^: CBT-I was administered through six weekly 50-min individual treatment sessions by a professional. VCBT-I ^13^: CBT-I was provided through self-administered 60-min weekly videos and weekly booklets for 6 weeks.	No Treatment	No treatment	Insomnia Severity Index Baseline PCBT-I: 14.0 VCBT-I: 14.5 No Treatment Control: 14.2 Post-Treatment PCBT-I: 5.9 VCBT-I: 8.3 No Treatment Control: 11.2	15.70%	CBT administered via video and by a professional were more effective than a no treatment control; however, professional administered CBT was more effective than video administered CBT.
Zachariae 2018, Denmark [50]	Randomized Controlled Trial	iCBT-I ^14^ was self-administered through 6 45–60 min cores across 6–9 weeks.	No Treatment	Waitlist Control	Insomnia Severity Index Baseline iCBT: 14.9 (4.8) Waitlist Control: 14.7 (4.5) Post-Treatment iCBT: 7.1 (4.4) Waitlist Control: 12.8 (5.3) Sleep Quality (PSQI) Baseline iCBT: 10.2 (3.6) Waitlist Control: 10.2 (3.0) Post-Treatment iCBT: 6.5 (2.8) Waitlist Control: 9.3 (3.4)	16.47%	iCBT-I is more effective than a waitlist control at improving sleep.
Brief Behavioral Interventions							
Dean 2020, United States [51]	Pilot Feasibility Study	Brief Behavioral Treatment for Insomnia (BBTI) ^15^ was delivered through 4 weekly sessions (2 in-person and 2 telephone) with 2 phone calls weekly for 2 weeks post-intervention.	Placebo	Healthy Eating Program consisting of 45-min educational sessions through 4 weekly sessions (2 in-person and 2 telephone) with 2 phone calls weekly for 2 weeks post-intervention.	Sleep Diary Sleep Efficiency Baseline BBTI: 72.04 (17.63) Healthy Eating Control: 73.00 (12.55) Post-Treatment BBTI: 85.21 (20.54) Healthy Eating Control: 79.70 (10.21)	25.00%	Brief Behavioral Therapy for Insomnia was more effective than a healthy eating control at improving sleep efficiency.
Palesh 2020, United States [52]	Pilot Randomized Controlled Trial	Brief Behavioral Therapy for Cancer-Related Insomnia (BBT-CI) ^16^ for 6 weeks was provided through one 60-min face-to-face session, four 15-min phone calls, and a second 60-min face-to-face “booster” session occurring 2 or 3 weeks following the initial session.	Placebo	The Control Condition consisted of a pamphlet that included sleep hygiene instructions recommended by the National Sleep Foundation that were general in nature but did not contain information that would be considered active components such as sleep restriction.	Insomnia Severity Index Baseline: BBT-CI: 14.20 (5.87) Sleep Hygiene Control: 12.74 (5.67) Post-Treatment BBT-CI: 8.185 Sleep Hygiene Control: 10.916	54.05%	Brief Behavioral Therapy for Cancer Related Insomnia is more effective than a Sleep Hygiene control at improving insomnia.
Progressive Muscle Relaxation							
Sari 2024, Turkey [53]	Randomized Controlled Trial	PMRE ^17^ was asked to be applied twice a day, before bedtime and at any convenient time during the day, for 8 weeks.	Placebo	Routine Care	PSQI Sleep Quality Baseline PMRE: 2.21 (0.54) Usual Care Control: 1.94 (0.73) Post-Treatment PMRE: 0.91 (0.57) Usual Care Control: 1.77 (0.65)	13.75%	Progressive Muscle Relaxation was more beneficial than the Routine Care control at improving sleep.
Turan 2024, Turkey [65]	Randomized Controlled Trial	Progressive muscle relaxation exercises were self-administered via audio for 8 weeks, every day of the week, for approximately 30 min each session, for a total of 56 sessions.	No Treatment	Waitlist Control	PSQI Sleep Quality Baseline: PMR ^17^: 1.35 (0.68) No Treatment Control: 1.57 (0.65) Post-Treatment PMR: 1.16 (0.5) No Treatment Control: 1.59 (0.5)	0%	Progressive Muscle Relaxation was more effective than a waitlist control in improving sleep quality.
Benson Relaxation Technique							
Chabok 2023, Iran [54]	Randomized Clinical Trial	BRT ^18^ was self-administered twice a day (preferably in the morning and in the afternoon) for 15 min each time for a period of 2 months at home.	No Treatment	No intervention	Sleep Quality Baseline BRT: 9.25 (2.50) No Treatment Control: 8.47 (2.13) Post-Treatment BRT: 6.63 (1.92) No Treatment Control: 8.41 (2.15)	0%	The Benson Relaxation Technique was effective at improving sleep quality compared to a no treatment control.
Mindfulness-Based Stress Reduction							
Garland 2014, Canada [55]	Randomized Partially-Blinded Non-Inferiority Trial	MBSR ^19^ was delivered in groups of 15–20 patients across 8 weekly 90-min sessions. Plus one 6-hour weekend silent retreat.	Active Comparator	CBT-I was delivered to groups of six to 10 individuals over the course of eight, weekly, 90-min sessions.	Insomnia Severity Index Baseline CBT-I: 17.75 (0.58) MBSR: 16.89 (0.65) Post-Treatment CBT-I: 8.20 (0.58) MBSR: 11.86 (0.65)	36.94%	Mindfulness-Based Stress Reduction and CBT-I were effective at improving sleep, though CBT-I was associated with more rapid and durable outcomes compared to MBSR.
Home-Based Psychological Nursing							
Li 2021, China [56]	Randomized Trial	Five weeks of home-based psychological nursing interventions, including health education, surgical information, family communication establishment, post-surgery topics, facilitated conversations with family about hypopharyngeal carcinoma and precautions after surgery.	Placebo	Standard Nursing	PSQI Global Sleep Quality Baseline Psychological Nursing: 8.51 (0.66) Standard Nursing: 8.55 (0.66) Post-Treatment Psychological Nursing: 7.15 (0.43) Standard Nursing: 7.48 (0.57)	0%	Home-Based Psychological Nursing was more effective at improving sleep quality compared to the standard nursing group.
Mindfulness-Based Cognitive Therapy							
Zhao 2020, China [57]	Randomized Controlled Trial	The MBCT–I ^20^ protocol was delivered to groups of 8–10 participants over 6 weekly 90–min sessions, for nine contact hours. Participants were instructed to maintain their own personal practice of mindfulness meditation for 20–40 min per day between sessions.	No Treatment	Waitlist Control	Insomnia Severity Index Baseline MBCT-I: 15.93 (2.90) Waitlist Control: 16.16 (2.77) Post-Treatment MBCT-I: 12.65 (2.86) Waitlist Control: 15.48 (2.93)	7.35%	Mindfulness-Based Cognitive Therapy was more effective than a waitlist control at improving insomnia.
Mindfulness Meditation and Mind-Body Bridging							
Nakamura 2013, United States [58]	Pilot Randomized Controlled Trial	Mindfulness meditation was administered in 3 weekly group meetings with home practice. Mind-body bridging was administered in 3 weekly group sessions.	Placebo	The SHE ^21^ intervention consisted of educational classes informing patients about how to change habits to improve sleep, and what to do if they had concerns about sleep quality.	Medical Outcomes Study Sleep Scale Baseline MBB ^22^: 58.01 (14.64) MM ^23^: 63.33 (12.70) SHE: 54.94 (18.31) Post-Treatment Mean (95% CI) MBB: 32.94 (26.37–39.50) MM: 41.29 (34.97–47.61) SHE: 50.04 (43.27–56.80)	3.51%	Both Mindfulness Meditation and Mind-Body Bridging were more effective in reducing sleep disturbance compared to the sleep hygiene control group.

^1^ CBT-I = Cognitive Behavioral Therapy for Insomnia, ^2^ mCBT-I = Minimal Cognitive Behavioral Therapy for Insomnia, ^3^ dCBT = Digital Cognitive Behavioral Therapy, ^4^ PSQI = Pittsburgh Sleep Quality Index, ^5^ IQR = Interquartile Range, ^6^ TAU = Treatment as Usual, ^7^ TCC = Tai Chi Chih, ^8^ BPT = Behavioral Placebo Treatment, ^9^ WASO = Wake After Sleep Onset, ^10^ TST = Total Sleep Time, ^11^ CBTi.p. = Cognitive Behavioral Therapy for Insomnia and Pain, ^12^ PCBT-I = Professional-Delivered Cognitive Behavioral Therapy for Insomnia, ^13^ VCBT-I = Video-Delivered Cognitive Behavioral Therapy, ^14^ iCBT-I = Internet-Delivered Cognitive Behavioral Therapy, ^15^ BBT-I = Brief Behavioral Therapy for Insomnia, ^16^ BBT-CI = Brief Behavioral Therapy for Cancer-Related Insomnia, ^17^ PMRE/PMR = Progressive Muscle Relaxation, ^18^ BRT = Benson Relaxation Technique, ^19^ MBSR = Mindfulness-Based Stress Reduction, ^20^ MBCT-I = Mindfulness-Based Cognitive Therapy for Insomnia, ^21^ SHE = Sleep Hygiene Education, ^22^ MBB = Mind-Body Bridging, ^23^ MM = Mindfulness Meditation.

## Supplementary Materials

The following supporting information can be downloaded at: https://www.mdpi.com/article/10.3390/cancers16223850/s1, **Table S1.** Ovid MEDLINE search strategy. **Table S2.** Ovid Embase search strategy. **Table S3.** Ovid PsycInfo search strategy. **Table S4.** EBSCO CINAHL Plus with Full Text search strategy. **Table S5.** Wiley Cochrane Library search strategy.
