# Supplementary material for: Psychological Interventions for Insomnia in Patients with Cancer: A Scoping Review
Source: Cancers (Basel). 2024 Nov 16;16(22):3850. doi: 10.3390/cancers16223850 (PMC11593114; doi:10.3390/cancers16223850)
Supplement: Supplementary file 1 [file cancers-16-03850-s001.zip › cancers-3298621-supplementary.pdf]

Supplemental Tables

Supplementary Table S1. Ovid MEDLINE search strategy.

#	Search
1	Neoplasms/
2	exp *Neoplasms/
3	(cancer* or carcinom* or tumor* or tumour* or neoplas* or myeloma* or leuk?emia* or lymphoma* or sarcoma* or melanoma* or osteosarcoma* or adenocarcinoma* or oncology).ti,kf.
4	(cancer* or carcinom* or tumor* or tumour* or neoplas* or myeloma* or leuk?emia* or lymphoma* or sarcoma* or melanoma* or osteosarcoma* or adenocarcinoma* or oncology).ab. /freq=2
5	1 or 2 or 3 or 4 [Cancer]
6	limit 5 to (English language and yr="2000 -Current")
7	(animals not (humans and animals)).sh.
8	6 not 7
9	(mice or mouse or murine or rat or rats or rodent or dog or dogs or cells or "in vitro" or "cell line*" or preclinical or "pre-clinical").ti.
10	8 not 9 [Remove animal study]
11	(review not systematic review).pt.
12	case report*.pt,ti.
13	(case series or retrospective study).ti.
14	(comment or news or editorial).pt.
15	11 or 12 or 13 or 14
16	10 not 15 [Remove review, case reports, case series, retrospective study]
17	exp Sleep/
18	exp Sleep–Wake Disorders/
19	exp "Sleep Initiation and Maintenance Disorders"/
20	(insomnia or wakefulness or sleeplessness).ti,ab,kf.
21	(sleep adj2 (deprivation or disturbance* or disorder* or disruption* or latency or efficiency or initiation or duration or quality)).ti,ab,kf.
22	(daytime adj2 (sleepiness* or somnolence)).ti,ab,kf.
23	("total sleep time" or "poor sleep").ti,ab,kf.
24	or/17-23 [Sleep Disorders]
25	16 and 24 [Cancer + Sleep Disorder/Insomnia]
26	randomized controlled trial.pt.
27	controlled clinical trial.pt.
28	random allocation.sh.
29	double-blind method.sh.
30	single-blind method.sh.
31	(randomized or randomized or randomly).ti,ab.
32	clinical trial.pt.
33	Clinical Trials as Topic/
34	((singl* or doubl* or tripl* or trebl*) adj10 (blind* or mask*)).ti,ab.
35	placebo*.ti,ab.
36	groups.ab.
37	drug therapy.fs.
38	(trial or (phase adj3 study)).ti.
39	Research Design/
40	control groups/
41	Prospective Studies/
42	prospective*.ti,ab,kf.

43	("phase II*" or "phase 2*" or "phase III" or "phase 3").ti,ab,kf.
44	exp "Surveys and Questionnaires"/
45	(Survey* or Questionnaire*).ti,ab.
46	or/26-45 [Study design: clinical trial/prospective study]
47	25 and 46 [Cancer + Sleep Disorder + Clinical Trials/Prospective study]
48	exp Cognitive Behavioral Therapy/
49	((cognitive or behavio?ral) adj3 (therap* or intervention* or psychotherap*)).ti,ab,kf.
50	(CBT or CBT-I or "e-CBT-I").ti.
51	exp Psychotherapy/
52	Sleep Hygiene/
53	exp Mind-Body Therapies/
54	Mindfulness/
55	exp Massage/
56	massage.ti,ab,kf.
57	Meditation.ti,ab,kf.
58	Weighted blanket*.ti,ab,kf.
59	("Sleep hygiene" or relaxation or Mindfulness or psychoeducational or psychotherapy or "Behavio?ral modification*").ti,ab,kf.
60	exp Counseling/ or (psychological adj3 counseling).ti,ab.
61	"Stimulus control".ti,ab,kf.
62	"Sleep restriction".ti,ab,kf.
63	exp Desensitization, Psychologic/ or "systematic desensitization".ti,ab,kf.
64	exp Hypnosis/
65	Hypnosis.ti,ab,kf.
66	exp Biofeedback, Psychology/
67	biofeedback.ti,ab,kf.
68	"Paradoxical intention".ti,ab,kf.
69	"Progressive muscle relaxation".ti,ab,kf.
70	"Autogenic training".ti,ab,kf.
71	"Body scan".ti,ab,kf.
72	((guided or psychotherap*) adj3 imagery).ti,ab,kf.
73	(Naps or Yoga or "Tai Chi" or "Tai Ji" or Qigong or "Qi Gong").ti,ab,kf.
74	exp Phototherapy/
75	light therap*.ti,ab,kf.
76	exp Musculoskeletal Manipulations/
77	reflexology.ti,ab,kf.
78	exp Exercise/ or Exercise.ti,kf.
79	exp Acupuncture Therapy/
80	Acupuncture.ti,ab,kf.
81	Acupressure.ti,ab,kf.
82	Moxibustion.ti,ab,kf.
83	("non pharmacologic*" or "nonpharmacologic*").ti,ab,kf.
84	Music Therapy/
85	music therapy.ti,ab,kf.
86	exp Melatonin/
87	Melatonin*.ti,ab,kf.
88	melatonin agonist*.mp.
89	Agomelatine*.mp.
90	Ramelteon*.mp.
91	Tasimelteon*.mp.
92	exp Benzodiazepines/
93	Benzodiazepine*.mp.

94	Amitriptyline*.mp.
95	Mirtazapine*.mp.
96	(Eszopiclone* or Lunesta*).mp.
97	(zaleplon* or sonata*).mp.
98	(zolpidem or Ambien* or Edluar* or Zolpimist).mp.
99	z-drug*.ti,ab.
100	(TCH or CBD).ti,ab.
101	cannabis*.mp.
102	exp Cannabinoids/
103	(Cannabinoid* or Cannabidiol*).mp.
104	Semen Ziziphi Spinosae.ti,ab,kf.
105	Ziziphus/
106	Ziziph*.mp.
107	Jujuboside A*.mp.
108	Valerian/
109	Valerian*.mp.
110	valerenic acid*.mp.
111	Lettuce/
112	(Lactuca sativa* or "lettuce seed*").mp.
113	Passiflora/
114	Passiflora Incarnata*.mp.
115	(passion flower* or passionflower*).mp.
116	exp Eschscholzia/
117	California poppy.mp.
118	Eschscholzia*.mp.
119	methysticum*.mp.
120	Kava/
121	("Piper methysticum*" or kava).mp.
122	exp Scutellaria/
123	Scutellaria*.mp.
124	skullcap.mp.
125	Pramipexole*.mp.
126	(("non-Benzodiazepine" or nonbenzodiazepine) adj3 Hypnotics).ti,ab,kf.
127	Orexin Receptor Antagonists/
128	orexin receptor antagonist*.ti,ab,kf.
129	suvorexant*.mp.
130	doxepin.mp.
131	exp Antidepressive Agents/
132	(antidepressant* or "Antidepressive Agent*" or "Antidepressive drug*").ti,ab,kf.
133	exp Diphenhydramine/
134	Diphenhydramine*.mp.
135	Doxylamine*.mp.
136	Temazepam/
137	temazepam*.mp.
138	exp Plants, Medicinal/
139	Ashwagandha*.mp.
140	or/48-139 [Interventions: nonpharmacological#48-#85, pharmacological#86-#139]
141	47 and 140 [Cancer + Sleep Disorder + Clinical Trials/Prospective study + Interventions]
142	sleep*.ti,ab,kf,sh,hw.
143	20 or 22 or 142 [further restriction by sleep/insomnia]
144	141 and 143

Supplementary Table S2. Ovid Embase search strategy

#	Search
1	exp malignant neoplasm/
2	(cancer* or carcinom* or tumor* or tumour* or neoplas* or myeloma* or leuk?emia* or lymphoma* or sarcoma* or melanoma* or osteosarcoma* or adenocarcinoma* or oncology).ti,kf.
3	(cancer* or carcinom* or tumor* or tumour* or neoplas* or myeloma* or leuk?emia* or lymphoma* or sarcoma* or melanoma* or osteosarcoma* or adenocarcinoma* or oncology).ab. /freq=2
4	1 or 2 or 3 [Cancer]
5	limit 4 to (English language and yr="2000 -Current")
6	(Nonhuman/ or ANIMAL/ or Animal Experiment/) not Human/
7	5 not 6
8	(mice or mouse or murine or rat or rats or rodent or dog or dogs or cells or "in vitro" or "cell line*" or preclinical or "pre-clinical").ti.
9	7 not 8 [Remove animal study]
10	limit 9 to "systematic review"
11	review.pt.
12	9 not 11
13	10 or 12
14	case study/
15	case report*.jx,ti.
16	(case series or retrospective study).ti.
17	(conference abstract or editorial or preprint or note).pt.
18	14 or 15 or 16 or 17
19	13 not 18 [Remove review, case reports, case series, retrospective study, conference abstract, preprint, etc.]
20	exp sleep/
21	exp sleep disorder/
22	exp sleep parameters/
23	(insomnia or wakefulness or sleeplessness).ti,ab,kf.
24	(sleep adj2 (deprivation or disturbance* or disorder* or disruption* or latency or efficiency or initiation or duration or quality)).ti,ab,kf.
25	(daytime adj2 (sleepiness* or somnolence)).ti,ab,kf.
26	("total sleep time" or "poor sleep").ti,ab,kf.
27	or/20-26 [Sleep Disorders]
28	19 and 27 [Cancer + Sleep Disorder/Insomnia]
29	Randomized Controlled Trial/
30	Controlled clinical trial/
31	Double Blind Procedure/
32	Single Blind Procedure/
33	exp randomization/
34	prospective study/
35	(randomized or randomized or randomly).ti,ab.
36	clinical trial/
37	Controlled Study/
38	experimental design/
39	((singl* or doubl* or tripl* or trebl*) adj5 (blind* or mask*)).ti,ab.
40	placebo*.ti,ab.
41	(trial or (phase adj3 study)).ti.
42	(trial or study).ti,ab. and groups.ab.
43	drug therapy.fs.
44	("phase 2*" or "phase 3*" or "phase II*" or phase III*).ti.
45	prospective*.ti,ab,kf.

46	exp questionnaire/
47	(Survey* or Questionnaire*).ti,ab.
48	or/29-47 [Study design: clinical trial/prospective study; survey]
49	28 and 48 [Cancer + Sleep Disorder + Clinical Trials/Prospective study; survey]
50	exp cognitive behavioral therapy/
51	((cognitive or behavio?ral) adj3 (therap* or intervention* or psychotherap*)).ti,ab.
52	(CBT or CBT-I or "e-CBT-I").ti.
53	exp psychotherapy/
54	sleep hygiene/
55	exp *alternative medicine/
56	exp mindfulness/
57	massage/
58	("Sleep hygiene" or relaxation or Mindfulness* or massage or psychoeducational or psychotherapy or "behavio?ral modification*").ti,ab.
59	exp meditation/
60	Meditation.ti,ab.
61	Weighted blanket*.ti,ab.
62	psychological counseling/
63	psychological counseling.ti,ab.
64	"Stimulus control".ti,ab.
65	"Sleep restriction".ti,ab.
66	systematic desensitization/
67	systematic desensitization.ti,ab.
68	hypnosis/
69	Hypnosis.ti,ab.
70	exp biofeedback/
71	biofeedback.ti,ab.
72	"Paradoxical intention".ti,ab.
73	"Progressive muscle relaxation".ti,ab.
74	"Autogenic training".ti,ab.
75	"Body scan".ti,ab.
76	((guided or psychotherap*) adj3 imagery).ti,ab.
77	"Autogenic training".ti,ab.
78	((guided or psychotherap*) adj3 imagery).ti,ab.
79	(Naps or Yoga or "Tai Chi" or "Tai Ji" or Qigong or "Qi Gong").ti,ab.
80	("light therap*" or phototherapy).ti,ab.
81	exp *exercise/ or Exercise.ti.
82	reflexology/ or reflexology.ti,ab.
83	exp *acupuncture/
84	exp Acupuncture Therapy/
85	moxibustion/
86	(Acupuncture or Acupressure or Moxibustion).ti,ab.
87	music therapy.ti,ab.
88	("non pharmacologic*" or "nonpharmacologic*").ti,ab.
89	melatonin/dt [Drug Therapy]
90	Melatonin*.ti,ab.
91	melatonin agonist*.mp.
92	(Agomelatine* or Ramelteon* or Tasimelteon*).ti,ab,du,dy,tn.
93	Benzodiazepine*.mp.
94	Amitriptyline*.mp.
95	Mirtazapine*.mp.
96	(Eszopiclone* or Lunesta*).mp.

97	(zaleplon* or sonata*).mp.
98	(zolpidem or Ambien* or Edluar* or Zolpimist).mp.
99	z-drug*.ti,ab.
100	(TCH or CBD).ti,ab.
101	cannabis*.ti,ab.
102	(Cannabinoid* or Cannabidiol*).ti,ab.
103	Semen Ziziphi Spinosae.ti,ab.
104	Ziziph*.mp.
105	Jujuboside A*.mp.
106	Valerian*.mp.
107	valerenic acid*.mp.
108	(Lactuca sativa* or "lettuce seed*").mp.
109	Passiflora Incarnata*.mp.
110	(passion flower* or passionflower*).mp.
111	(Eschscholzia or "California poppy").mp.
112	methysticum*.mp.
113	("Piper methysticum*" or kava).mp.
114	Scutellaria*.mp.
115	skullcap.mp.
116	Pramipexole*.mp.
117	(("non-Benzodiazepine" or nonbenzodiazepine) adj3 Hypnotics).ti,ab,kf.
118	exp orexin receptor antagonist/
119	orexin receptor antagonist*.ti,ab,kf.
120	suvorexant*.mp.
121	doxepin.mp.
122	exp antidepressant agent/dt [Drug Therapy]
123	antidepressant*.ti,ab.
124	Diphenhydramine*.mp.
125	Doxylamine*.mp.
126	temazepam*.mp.
127	Ashwagandha*.mp.
128	or/50-127 [Interventions: nonpharmacological#50-#88, pharmacological#89-#127]
129	49 and 128 [Cancer + Sleep Disorder + Clinical Trials/Prospective study + Interventions]
130	sleep*.ti,ab,kf,sh,hw.
131	(insomnia or wakefulness or sleeplessness or sleepiness* or somnolence).ti,ab,kf.
132	130 or 131
133	129 and 132 [further restriction by sleep/insomnia]

Supplementary Table S3. Ovid PsycInfo search strategy

#	**Search**
1	exp Neoplasms/
2	(cancer* or carcinom* or tumor* or tumour* or neoplas* or myeloma* or leuk?emia* or lymphoma* or sarcoma* or melanoma* or osteosarcoma* or adenocarcinoma* or oncology).ti,ab,id.
3	1 or 2
4	limit 3 to (English language and yr="2000 -Current")
5	(mice or mouse or murine or rat or rats or rodent or dog or dogs or cells or "in vitro" or "cell line*" or preclinical or "pre-clinical").ti.
6	4 not 5 [Remove animal study]
7	limit 6 to "0800 literature review"
8	limit 6 to "0830 systematic review"

9	limit 6 to reviews
10	7 or 9
11	6 not 10
12	8 or 11
13	("case report*" or case series or "retrospective study").ti.
14	12 not 13 [Remove review, case reports, case series, retrospective study]
15	exp sleep/
16	sleep deprivation/
17	exp sleep–wake disorders/
18	sleep quality/
19	sleepiness/
20	(insomnia or wakefulness or sleeplessness).ti,ab,id,hw.
21	(sleep adj2 (deprivation or disturbance* or disorder* or disruption* or latency or efficiency or initiation or duration or quality)).ti,ab,id,hw.
22	(daytime adj2 (sleepiness* or somnolence)).ti,ab,id,hw.
23	("total sleep time" or "poor sleep").ti,ab,id,hw.
24	or/15-23 [sleep disorders]
25	12 and 24 [Cancer + Sleep Disorder/Insomnia]
26	exp Cognitive Behavior Therapy/
27	((cognitive or behavio?ral) adj3 (therap* or intervention* or psychotherap*)).ti,ab,id,hw.
28	(CBT or CBT-I or "e-CBT-I").ti.
29	exp psychotherapy/
30	exp Mind Body Therapy/
31	mindfulness/
32	mindfulness-based interventions/
33	meditation/
34	("Sleep hygiene" or relaxation or Mindfulness or psychoeducational or psychotherapy or "Behavio?ral modification*").ti,ab,id,hw.
35	exp Massage/
36	exp Psychotherapeutic Techniques/
37	(massage or "weighted blanket*").ti,ab.
38	exp Psychotherapeutic Counseling/
39	((psychological or psychotherapeutic) adj3 counseling).ti,ab.
40	stimulus control/
41	"Stimulus control".ti,ab.
42	"Sleep restriction".ti,ab,id,hw.
43	exp Systematic Desensitization Therapy/
44	"systematic desensitization".ti,ab,id,hw.
45	hypnosis/
46	Hypnosis.ti,ab,id,hw.
47	exp Biofeedback/
48	biofeedback.ti,ab,id,hw.
49	exp Paradoxical Techniques/
50	"Paradoxical intention".ti,ab,id,hw.
51	exp Progressive Relaxation Therapy/
52	"Autogenic training".ti,ab,id,hw.
53	"Body scan".ti,ab.
54	((guided or psychotherap*) adj3 imagery).ti,ab,id,hw.
55	(Naps or Yoga or "Tai Chi" or "Tai Ji" or Qigong or "Qi Gong").ti,ab,id,hw.
56	exp Phototherapy/
57	light therap*.ti,ab,id,hw.
58	exp alternative medicine/

59	reflexology.ti,ab,id,hw.
60	(Acupuncture or Acupressure or Moxibustion).ti,ab,id,hw.
61	exp Exercise/ or exercise.ti,id,hw.
62	("non pharmacologic*" or "nonpharmacologic*").ti,ab,id,hw.
63	music therapy/
64	music therapy.ti,ab,id,hw.
65	exp Melatonin/
66	melatonin agonist*.mp.
67	melatonin*.ti,ab,id,hw.
68	(Agomelatine* or Ramelteon* or Tasimelteon*).mp.
69	exp benzodiazepines/
70	(Benzodiazepine* or Amitriptyline* or Mirtazapine*).mp.
71	(Eszopiclone* or Lunesta* or zaleplon* or sonata* or zolpidem or Ambien* or Edluar* or Zolpimis).mp.
72	z-drug*.ti,ab.
73	(TCH or CBD).ti,ab.
74	exp cannabis/
75	cannabis*.ti,ab,id,hw.
76	cannabinoids/
77	(Cannabinoid* or Cannabidiol*).mp.
78	exp "Medicinal Herbs and Plants"/
79	Ziziph*.mp.
80	Jujuboside A*.mp.
81	Valerian*.mp.
82	valerenic acid*.mp.
83	(Lactuca sativa* or "lettuce seed*").mp.
84	(Passiflora* or "passion flower*" or passionflower*).mp.
85	(Eschscholzia* or "California poppy").mp.
86	(methysticum* or kava).mp.
87	(scutellaria* or skullcap).mp.
88	Pramipexole*.mp.
89	(("non-Benzodiazepine" or nonbenzodiazepine) adj3 Hypnotics).mp.
90	orexin/
91	orexin receptor antagonist*.mp.
92	suvorexant*.mp.
93	doxepin*.mp.
94	exp Antidepressant Drugs/
95	antidepressant*.mp.
96	Diphenhydramine*.mp.
97	Doxylamine*.mp.
98	exp Hypnotic Drugs/
99	temazepam*.mp.
100	Ashwagandha*.mp.
101	exp Drug Therapy/
102	or/26-101
103	25 and 102
104	limit 25 to "therapy (best balance of sensitivity and specificity)"
105	103 or 104
106	sleep*.mp.
107	(insomnia or wakefulness or sleeplessness or sleepiness* or somnolence).mp.
108	106 or 107
109	105 and 108

Supplementary Table S4. EBSCO CINAHL Plus with Full Text search strategy

**S94**	**S87 AND S93**
S93	S88 OR S89 OR S90 OR S91 OR S92
S92	TX (survey* or questionnaire* or prospective*) OR AB (survey* or questionnaire* or prospective*)
S91	(MH "Questionnaires+")
S90	(MH "Surveys+")
S89	(MH "Prospective Studies+")
S88	(randomized controlled trials OR MH double-blind studies OR MH single-blind studies OR MH random assignment OR MH pretest-posttest design OR MH cluster sample OR TI (randomized OR randomized) OR AB (random*) OR TI (trial) OR (MH (sample size) AND AB (assigned OR allocated OR control)) OR MH (placebos) OR PT (randomized controlled trial) OR AB (control W5 group) OR MH (crossover design) OR MH (comparative studies) OR AB (cluster W3 RCT)) NOT ((MH animals+ OR MH animal studies OR TI animal model*) NOT MH human)
S87	S83 AND S86
S86	S81 AND S85
S85	S82 OR S84
S84	(MM "Neoplasms+")
S83	TI ((sleep* or insomnia or wakefulness or sleeplessness or sleepiness* or somnolence)) OR AB ((sleep* or insomnia or wakefulness or sleeplessness or sleepiness* or somnolence)) OR MW ((sleep* or insomnia or wakefulness or sleeplessness or sleepiness* or somnolence)) OR SU ((sleep* or insomnia or wakefulness or sleeplessness or sleepiness* or somnolence))
S82	TI ((cancer* or carcinom* or tumor* or tumour* or neoplas* or myeloma* or leuk*emia* or lymphoma* or sarcoma* or melanoma* or osteosarcoma* adenocarcinoma* or oncology)) OR MW ((cancer* or carcinom* or tumor* or tumour* or neoplas* or myeloma* or leuk*emia* or lymphoma* or sarcoma* or melanoma* or osteosarcoma* adenocarcinoma* or oncology)) OR MJ ((cancer* or carcinom* or tumor* or tumour* or neoplas* or myeloma* or leuk*emia* or lymphoma* or sarcoma* or melanoma* or osteosarcoma* adenocarcinoma* or oncology)) OR SU ((cancer* or carcinom* or tumor* or tumour* or neoplas* or myeloma* or leuk*emia* or lymphoma* or sarcoma* or melanoma* or osteosarcoma* adenocarcinoma* or oncology))
S81	S13 AND S78
S80	S13 AND S78
S79	S13 AND S78
S78	S14 OR S15 OR S16 OR S17 OR S18 OR S19 OR S20 OR S21 OR S22 OR S23 OR S24 OR S25 OR S26 OR S27 OR S28 OR S29 OR S30 OR S31 OR S32 OR S33 OR S34 OR S35 OR S36 OR S37 OR S38 OR S39 OR S40 OR S41 OR S42 OR S43 OR S44 OR S45 OR S46 OR S47 OR S48 OR S49 OR S50 OR S51 OR S52 OR S53 OR S54 OR S55 OR S56 OR S57 OR S58 OR S59 OR S60 OR S61 OR S62 OR S63 OR S64 OR S65 OR S66 OR S67 OR S68 OR S69 OR S70 OR S71 OR S72 OR S73 OR S74 OR S75 OR S76 OR S77
S77	(MH "Plants, Medicinal+")
S76	TI Ashwagandha OR AB Ashwagandha
S75	TI ((Doxylamine* OR Temazepam*)) OR AB ((Doxylamine* OR Temazepam*))
S74	(MH "Temazepam")
S73	(MH "Diphenhydramine+")
S72	TI (("Antidepressive Agent*" OR "Antidepressive drug*") OR AB (("Antidepressive Agent*" OR "Antidepressive drug*")
S71	TI antidepressant* OR AB antidepressant*
S70	(MH "Antidepressive Agents+")

S69	TI (suvorexant* OR doxepin) OR AB (suvorexant* OR doxepin)
S68	TI "Orexin Receptor Antagonist*" OR AB "Orexin Receptor Antagonist*"
S67	TI ((("non-Benzodiazepine" or nonbenzodiazepine) N3 Hypnotics)) OR AB ((("non-Benzodiazepine" or nonbenzodiazepine) N3 Hypnotics))
S66	TI (Scutellaria* OR Skullcap OR Pramipexole*) OR AB (Scutellaria* OR Skullcap OR Pramipexole*)
S65	TI ("Piper methysticum*" or kava) OR AB ("Piper methysticum*" or kava)
S64	(MH "Kava Kava")
S63	TI ((Eschscholzia OR "California poppy")) OR AB ((Eschscholzia OR "California poppy"))
S62	TI (Passiflora OR passion flower* OR passionflower*) OR AB (Passiflora OR passion flower* OR passionflower*)
S61	(MH "Passionflower")
S60	TI ((Lactuca sativa* or "lettuce seed*")) OR AB ((Lactuca sativa* or "lettuce seed*"))
S59	TI (Valerian* OR "valerenic acid*") OR AB (Valerian* OR "valerenic acid*")
S58	(MH "Valerian")
S57	TI Jujuboside A* OR AB Jujuboside A*
S56	TI Ziziph* OR AB Ziziph*
S55	TI ((Cannabis* OR annabinoid* or Cannabidiol*)) OR AB ((Cannabis* OR annabinoid* or Cannabidiol*))
S54	(MH "Cannabinoids+")
S53	(MH "Cannabis+")
S52	TI (TCH or CBD) OR AB (TCH or CBD)
S51	TI z-drug* OR AB z-drug*
S50	TI ((Benzodiazepine* OR Amitriptyline* OR Mirtazapine* OR Eszopiclone* OR Lunesta* OR zaleplon* OR sonata* OR zolpidem or Ambien* or Edluar* or Zolpimist)) OR AB ((Benzodiazepine* OR Amitriptyline* OR Mirtazapine* OR Eszopiclone* OR Lunesta* OR zaleplon* OR sonata* OR zolpidem or Ambien* or Edluar* or Zolpimist))
S49	(MH "Antianxiety Agents, Benzodiazepine+")
S48	TI (Agomelatine* OR Ramelteon* OR Tasimelteon*) OR AB (Agomelatine* OR Ramelteon* OR Tasimelteon*)
S47	TI Melatonin OR AB Melatonin
S46	(MH "Melatonin")
S45	TI ("non pharmacologic*" or "nonpharmacologic*
S44	TI music therapy OR AB music therapy
S43	(MH "Music Therapy")
S42	TI (Acupuncture OR Acupressure OR Moxibustion) OR AB (Acupuncture OR Acupressure OR Moxibustion)
S41	(MH "Moxibustion")
S40	(MH "Acupuncture+")
S39	TI Exercise
S38	(MM "Exercise")
S37	TI reflexology OR AB reflexology
S36	(MH "Reflexology")
S35	TI light therap* OR AB light therap*
S34	(MH "Phototherapy+")
S33	TI ((Naps or Yoga or "Tai Chi" or "Tai Ji" or Qigong or "Qi Gong")) OR AB ((Naps or Yoga or "Tai Chi" or "Tai Ji" or Qigong or "Qi Gong"))
S32	TI (((guided or psychotherap*) N3 imagery)) OR AB (((guided or psychotherap*) N3 imagery))
S31	TI (("Hypnosis" OR Biofeedback OR "Paradoxical intention" OR "Progressive muscle relaxation" OR "Autogenic training" OR "Body scan")) OR AB

	(("Hypnosis" OR Biofeedback OR "Paradoxical intention" OR "Progressive muscle relaxation" OR "Autogenic training" OR "Body scan"))
S30	(MH "Autogenic Training (Iowa NIC)")
S29	(MH "Progressive Muscle Relaxation (Iowa NIC)")
S28	TI systematic desensitization OR AB systematic desensitization
S27	(MH "Desensitization, Psychologic+")
S26	TI "Sleep restriction" OR AB "Sleep restriction"
S25	TI "Stimulus control" OR AB "Stimulus control"
S24	TI psychological N3 counseling OR AB psychological N3 counseling
S23	TI (("Sleep hygiene" or relaxation or Mindfulness or psychoeducational or psychotherapy or "Behavio*ral modification*")) OR AB (("Sleep hygiene" or relaxation or Mindfulness or psychoeducational or psychotherapy or "Behavio*ral modification*"))
S22	TI (massage or meditation or "weighted blanket*") OR AB (massage or meditation or "weighted blanket*")
S21	(MH "Massage+")
S20	(MH "Mindfulness+")
S19	(MH "Mind Body Techniques+")
S18	(MH "Sleep Hygiene+")
S17	(MH "Psychotherapy+")
S16	TI (CBT or CBT-I or "e-CBT-I")
S15	TI (((cognitive or behavio*ral) N3 (therap* or intervention* or psychotherap*))) OR AB (((cognitive or behavio*ral) N3 (therap* or intervention* or psychotherap*)))
S14	(MH "Cognitive Therapy+")
S13	S5 AND S12
S12	S6 OR S7 OR S8 OR S9 OR S10 OR S11
S11	TI (("total sleep time" or "poor sleep")) OR AB (("total sleep time" or "poor sleep"))
S10	TI ((daytime N2 (sleepiness* or somnolence))) OR AB ((daytime N2 (sleepiness* or somnolence)))
S9	TI ((sleep N2 (deprivation or disturbance* or disorder* or disruption* or latency or efficiency or initiation or duration or quality))) OR AB ((sleep N2 (deprivation or disturbance* or disorder* or disruption* or latency or efficiency or initiation or duration or quality)))
S8	TI (insomnia or wakefulness or sleeplessness) OR AB (insomnia or wakefulness or sleeplessness)
S7	(MH "Sleep Disorders+")

Supplementary Table S5. Wiley Cochrane Library search strategy

#	**Search**
#1	MeSH descriptor: [Neoplasms] explode all trees
#2	(cancer* or carcinom* or tumor* or tumour* or neoplas* or myeloma* or leuk?emia* or lymphoma* or sarcoma* or melanoma* or osteosarcoma* or adenocarcinoma* or oncology):ti,ab,kw (Word variations have been searched)
#3	#1 or #2
#4	((mice or mouse or murine or rat or rats or rodent or dog or dogs or cells or "in vitro" or "cell line*" or preclinical or "pre-clinical")):ti (Word variations have been searched)
#5	#3 not #4
#6	MeSH descriptor: [Sleep] explode all trees
#7	MeSH descriptor: [Sleep Wake Disorders] explode all trees
#8	MeSH descriptor: [Sleep Initiation and Maintenance Disorders] explode all trees

#9	(insomnia or wakefulness or sleeplessness):ti,ab,kw (Word variations have been searched)
#10	((sleep near/2 (deprivation or disturbance* or disorder* or disruption* or latency or efficiency or initiation or duration or quality))):ti,ab,kw (Word variations have been searched)
#11	((daytime near/2 (sleepiness* or somnolence))):ti,ab,kw (Word variations have been searched)
#12	(("total sleep time" or "poor sleep")):ti,ab,kw (Word variations have been searched)
#13	{OR #6-#12}
#14	#5 AND #13
#15	MeSH descriptor: [Cognitive Behavioral Therapy] explode all trees
#16	(((cognitive or behavio?ral) NEAR/3 (therap* or intervention* or psychotherap*))):ti,ab,kw (Word variations have been searched)
#17	((CBT or CBT-I or "e-CBT-I")):ti (Word variations have been searched)
#18	MeSH descriptor: [Psychotherapy] explode all trees
#19	MeSH descriptor: [Sleep Hygiene] explode all trees
#20	MeSH descriptor: [Mind-Body Therapies] explode all trees
#21	MeSH descriptor: [Mindfulness] explode all trees
#22	MeSH descriptor: [Massage] explode all trees
#23	(("Sleep hygiene" or relaxation or mindfulness or psychoeducational or psychotherapy or "behavio?ral modification*" or massage or "weighted blanket*" or meditation)):ti,ab,kw (Word variations have been searched)
#24	MeSH descriptor: [Counseling] explode all trees
#25	(psychological near/3 counseling):ti,ab,kw (Word variations have been searched)
#26	MeSH descriptor: [Desensitization, Psychologic] explode all trees
#27	("stimulus control" or "Sleep restriction" or "systematic desensitization"):ti,ab,kw (Word variations have been searched)
#28	((Hypnosis or biofeedback or "Paradoxical intention" or "Progressive muscle relaxation" or "Autogenic training" or "Body scan")):ti,ab,kw (Word variations have been searched)
#29	(((guided or psychotherap*) Near/3 imagery)):ti,ab,kw (Word variations have been searched)
#30	((Naps or Yoga or "Tai Chi" or "Tai Ji" or Qigong or "Qi Gong")):ti,ab,kw (Word variations have been searched)
#31	MeSH descriptor: [Phototherapy] explode all trees
#32	(light therap*):ti,ab,kw (Word variations have been searched)
#33	MeSH descriptor: [Musculoskeletal Manipulations] explode all trees
#34	(reflexology):ti,ab,kw (Word variations have been searched)
#35	MeSH descriptor: [Exercise] explode all trees
#36	(exercise):ti,ab,kw (Word variations have been searched)
#37	MeSH descriptor: [Acupuncture Therapy] explode all trees
#38	(Acupuncture or Acupressure or Moxibustion):ti,ab,kw (Word variations have been searched)
#39	MeSH descriptor: [Music Therapy] explode all trees
#40	(music therapy):ti,ab,kw (Word variations have been searched)
#41	(("non pharmacologic*" or "nonpharmacologic*")):ti (Word variations have been searched)
#42	{OR #15-#41}
#43	#14 and #42
#44	MeSH descriptor: [Melatonin] explode all trees
#45	((Melatonin* or Agomelatine* or Ramelteon* or Tasimelteon*)):ti,ab,kw (Word variations have been searched)
#46	MeSH descriptor: [Benzodiazepines] explode all trees

#47	((Eszopiclone* or Lunesta* or zaleplon* or sonata* or zolpidem or Ambien* or Edluar* or Zolpimis or "z-drug*" or TCH or CBD)):ti,ab,kw (Word variations have been searched)
#48	MeSH descriptor: [Cannabis] explode all trees
#49	MeSH descriptor: [Cannabinoids] explode all trees
#50	((Cannabis* or Cannabinoid* or Cannabidiol*)):ti,ab,kw (Word variations have been searched)
#51	MeSH descriptor: [Plants, Medicinal] explode all trees
#52	MeSH descriptor: [Ziziphus] explode all trees
#53	MeSH descriptor: [Valerian] explode all trees
#54	MeSH descriptor: [Passiflora] explode all trees
#55	MeSH descriptor: [Eschscholzia] explode all trees
#56	MeSH descriptor: [Kava] explode all trees
#57	MeSH descriptor: [Scutellaria] explode all trees
#58	((Ziziph* or "jujuboside A*" or valerian* or "valerenic acid*" or "Lactuca sativa*" or "lettuce seed*" or Passiflora* or "passion flower*" or passionflower* or Eschscholzia* or "California poppy" or methysticum* or kava or scutellaria* or skullcap or Pramipexole*)):ti,ab,kw (Word variations have been searched)
#59	((("non-Benzodiazepine" or nonbenzodiazepine) NEAR/3 Hypnotics)):ti,ab,kw (Word variations have been searched)
#60	MeSH descriptor: [Orexin Receptor Antagonists] explode all trees
#61	(orexin receptor antagonist*):ti,ab,kw (Word variations have been searched)
#62	((suvorexant* OR doxepin)):ti,ab,kw (Word variations have been searched)
#63	MeSH descriptor: [Antidepressive Agents] explode all trees
#64	((antidepressant* OR "Antidepressive Agent*" OR "Antidepressive drug*")):ti,ab,kw (Word variations have been searched)
#65	MeSH descriptor: [Diphenhydramine] explode all trees
#66	MeSH descriptor: [Temazepam] explode all trees
#67	((Temazepam or Diphenhydramine* or Doxylamine*)):ti,ab,kw (Word variations have been searched)
#68	(Ashwagandha*):ti,ab,kw (Word variations have been searched)
#69	{OR #15-#68}
#70	#14 AND #69
#71	conference:pt
#72	#70 NOT #71
